# Transplantation of Human Gingiva-Derived Mesenchymal Stem Cells Ameliorates Neurotic Erectile Dysfunction in a Rat Model

**DOI:** 10.3389/fbioe.2021.630076

**Published:** 2021-06-21

**Authors:** Juekun Wu, Zehong Chen, Fuyan Zhong, Wende Yang, Xi Ouyang, Xiaolei Ma, Songguo Zheng, Hongbo Wei

**Affiliations:** ^1^Department of Thyroid and Breast Surgery, The Third Affiliated Hospital of Sun Yat-sen University, Guangzhou, China; ^2^Department of Gastrointestinal Surgery, The Third Affiliated Hospital of Sun Yat-sen University, Guangzhou, China; ^3^Central Laboratory, The Third Affiliated Hospital of Sun Yat-sen University, Guangzhou, China; ^4^Department of Gastrointestinal Surgery, The Second Affiliated Hospital of Nanchang University, Nanchang, China; ^5^Department of Internal Medicine, Ohio State University College of Medicine and Wexner Medical Center, Columbus, OH, United States

**Keywords:** human gingiva-derived MSCs, erectile dysfunction, cell therapy, mechanisms, cavernous nerve injury

## Abstract

Cavernous nerve injury (CNI) is the main cause of erectile dysfunction (ED) following pelvic surgery. Our previous studies have demonstrated that transplantation of different sources of mesenchymal stem cells (MSCs) was able to alleviate ED induced by CNI in rat models. However, little is known about the therapeutic effects of human gingiva-derived MSCs (hGMSCs) in CNI ED rats. Herein, we injected the hGMSCs around the bilateral major pelvic ganglia (MPG) in a rat model of CNI and evaluated their efficacy. The results showed that treatment of hGMSCs could significantly promote the recovery of erectile function, enhance smooth muscle and endothelial content, restore neuronal nitric oxide synthase (nNOS) expression, and attenuate cell apoptosis in penile tissue. Moreover, penile fibrosis was significantly alleviated after hGMSC administration. In addition, potential mechanism exploration indicated that hGMSCs might exert its functions via skewed macrophage polarity from M1 toward M2 anti-inflammatory phenotype. In conclusion, this study found that transplantation of hGMSCs significantly improved CNI-related ED, which might provide new clues to evaluate their pre-clinical application.

## Introduction

There are many causes of erectile dysfunction (ED), which include psychological factors, neurological disorders (such as multiple sclerosis, temporal lobe epilepsy, and cavernous nerve injury), and vasculogenic disorders (such as atherosclerosis, hypertension, and diabetes mellitus). Neurogenic sexual dysfunction makes up about 10–19% in all causes of erectile dysfunction. Neurotic erectile dysfunction is one of most important complications after radical prostatectomy and rectectomy, owing to intraoperative damage of the pelvic cavernous nerve (CN). It affects not only the physical but also mental health in postoperative patients. Despite the improvement of nerve-sparing techniques, the incidence of neurotic ED still has no substantial improvement. The incidences of ED range from 75 to 80% after pelvic surgery (Schauer et al., 2015). Moreover, large clinical trials showed that phosphodiesterase type 5 inhibitors (PDE5Is) are only partly effective in this specific kind of ED (Montorsi et al., 2014). Thus, it is urgently needed to find a new therapeutic strategy to improve the life quality of these special populations.

Studies have demonstrated that pelvic cavernous nerve injury (CNI) is the major cause of neurogenic ED (Capogrosso et al., 2016). On the one hand, CNI leads to reduced bioavailability of neuronal nitric oxide (NO). On the other hand, CNI combined with vascular injury might cause penile hypoxia and ischemia, which in turn results in the subsequent apoptosis of the corpus cavernosum. Furthermore, progressive fibrosis has been proved accelerating the progress of CNI-induced ED (Cho et al., 2011; Wan et al., 2018). Thus, how to reduce penile tissue atrophy and progressive fibrosis effectively should be considered as a major aspect in the therapy of CNI ED.

Stem cell-based therapy has been considered as a potential treatment method for several diseases. As one of the most eye-catching stem cells, mesenchymal stem cells (MSCs) are characterized by their regenerative ability, paracrine properties, and immunological modulations (Uccelli et al., 2008; Wang et al., 2014). Human gingival tissue-derived MSCs (hGMSCs), an optimized substitution for MSCs derived from other sources, is a population of MSCs isolated from human gingiva and presents many advantages over other source-derived MSCs. hGMSCs possess a more homogenous property, more retrievable source, and no tumorigenesis (Huang et al., 2017). Importantly, our previous studies have demonstrated the therapeutic and immunoregulatory effects of hGMSCs. For example, hGMSCs significantly reduced infiltration of Th1 and Th17 cells, whereas increased CD4^+^ Foxp3^+^ regulatory T cell (Treg) differentiation in lymph nodes resulted in attenuated T cell-mediated bone marrow failure and other inflammatory diseases (Chen et al., 2013; Su et al., 2015, 2017; Li et al., 2019; Luo Y. et al., 2019; Zhao et al., 2019). However, to the best of our knowledge, there is no study exploring whether hGMSCs have the ability to treat CNI ED.

In this study, we isolated hGMSCs from volunteers and made identification. Moreover, we established a CNI model in rats and firstly applied hGMSCs on this specific kind of ED model. The potential therapeutic effect of hGMSCs on CNI ED was explored and the underlying mechanism was discussed.

## Materials and Methods

### Cell Preparation

hGMSCs were isolated from the human gingiva tissue samples as previously described (Huang et al., 2017; Chen et al., 2019), which were collected at the Division of Dentistry in the Third Hospital at Sun Yat-sen University. The inclusion criteria were listed as follows: (1) male; (2) 19–30 years old; and (3) clinically healthy and underwent orthodontic treatment. There were 10 male volunteers who participated in the study, and the average age was 26. There are six gingival tissues collected from the upper and four from the lower gingiva. All the volunteers signed informed consent before collection. The study was approved by the medical ethics committees of the Third Hospital of Sun Yat-sen University.

Briefly, the volunteers received topical anesthesia by 1% lidocaine. The gingival tissues were then collected following routine dental procedures. Then, the gingival tissues were minced into 1–3-mm^2^ fragments immediately and treated with Dispase II (2 mg/ml) at 4°C overnight followed with digestion by collagenase IV (4 mg/ml) at 37°C for 2 h. After being filtered through a 40-μm cell strainer (Falcon), the dissociated cells were plated in 10-cm cell culture dishes with MEM alpha (Gibco) medium supplemented with 10% fetal bovine serum (Gibco), 100 U/ml penicillin, and 100 μg/ml streptomycin (Gibco) and incubated under standard conditions (humidified atmosphere, 37°C, 5% CO_2_). The non-adherent cells were removed after being cultured for 72 h. The remaining adherent cells were passaged and cultivated in a T75 cell culture flask. When the cells reached 75% density, they were digested and passaged. The cells were counted under a microscope and a total of 1 × 10^6^ cells were prepared for therapy in a single rat. The cells were used between three and five passages (Cho et al., 2011; Capogrosso et al., 2016; Wan et al., 2018).

### Characterization of hGMSCs

Flow cytometry was used to identify the characterization markers of the MSCs. hGMSCs were stained with CD1b, CD44, CD90, CD105, CD73, HLA-ABC, and HLA-DR (all from Abcam, Cambridge, United Kingdom) and then assessed by flow cytometry.

### Adipogenic Differentiation

To determine the potential for adipogenic differentiation of hGMSCs, the cells were subjected to adipogenic differentiation assays. The hGMSCs were cultured in 24-well plates, and the medium was changed with adipogenic differentiation medium when the cells reached 100% confluency. The medium contains 10% fetal bovine serum, 100 U/ml penicillin, 100 μg/ml streptomycin, 1% glutamine, 0.2% insulin, 0.1% IBMX, 0.1% rosiglitazone, and 0.1% dexamethasone. The medium was changed every 3 days, and the cells were stained by Oil Red O 2 weeks later. Human skin fibroblasts (HSFb) were used as controls.

### Osteogenic Differentiation

hGMSCs were counted and seeded in a 24-well plate with 8 × 10^3^ cells. The plates were covered with gelatin before use. When the cells reached 75% confluency, the medium was replaced by an osteogenic differentiation medium. The osteogenic differentiation medium contains 10% fetal bovine serum, 100 U/ml penicillin, 100 μg/ml streptomycin, 0.25 mg/ml amphotericin, 100 mg/ml kanamycin, 1% β-glycerophosphate, 10^–7^ M dexamethasone, and 0.2% ascorbate. The cells were cultured in standard conditions (humidified atmosphere, 37°C, 5% CO_2_). The medium was changed every 3 days, and the cells were stained by von Kossa 2 weeks later. HSFb were used as controls.

### Chondrogenic Differentiation

hGMSCs were counted and seeded in a 15-ml centrifuge tube with 4 × 10^5^ cells. Then, the cells in the centrifuge tube were cultured with a chondrogenic differentiation medium, which contains 0.3% ascorbate, 10^–7^ M dexamethasone, 0.1% sodium pyruvate, 0.1% proline, and 1% TGF-β3. The cap of the centrifuge tube was lightly loosened, and the cells were cultured in standard conditions (humidified atmosphere, 37°C, 5% CO_2_). The medium was changed every 3 days for 3 weeks, and the cartilage balls were fixed with 10% formalin. The specimens were made as tissue slice and stained by Alcian blue staining. HSFb were used as controls.

### Animal Model and Treatment

Twenty-four 8-week-old Sprague Dawley male rats (average weight 250 g) were purchased from Guangdong Medical Laboratory Animal Center (Guangdong, China). The rats were raised at the Experimental Animal Center of the Third Affiliated Hospital of Sun Yat-sen University and maintained in a 12-h light–dark cycle. All animals were acclimatized to the environment for 1 week at least before surgery.

The experiment was approved by the Animal Care and Use Subcommittee of The Third Affiliated Hospital of Sun Yat-sen University. All the rats received 2.5–3% isoflurane for anesthesia before surgery. For sham surgery, the rats received only laparotomy but no CN crush. For CNI surgery, for the purpose of exposing the major pelvic ganglia (MPG) and the cavernous nerve, we made a 5-cm incision in the middle hypogastric region of the rats. Then, we adopt a non-serrated hemostat (Karl Storz) to establish the CNI. The bilateral CNs, away from the MPG about 1 mm, were mechanically squeezed by hemostat for 2 min.

Twenty-four male SD rats (8 weeks of age) were randomly divided into three groups (eight rats in each group): sham surgery (sham group), CNI surgery and injected with 100 μl phosphate-buffered saline (PBS) around the MPG (PBS group), and CNI surgery and injected with hGMSCs (1 × 10^6^ cells in 100 μl PBS) around the MPG (hGMSC group). The size of the MPG was about 5 mm, and the injection around the MPG was bilateral injection, respectively, by using a 100-μl pipettor.

### Functional Evaluation

Two weeks after cells transplantation, erectile functions of SD rats were assessed. The penile and the MPG tissues were collected. The CN was electrically stimulated to assess erectile function. The rats were anesthetized with 2.5–3% isoflurane and cut open from the chest to the neck. The right carotid artery was fully displayed to measure the mean arterial pressure (MAP). A 24-gauge silicone rubber cannula was used to measure the MAP. The cavernous nerve was shown by exposing the prostate fully. The skin of the penis was stripped and the corpus cavernosa was exposed; then, a detection needle was implanted on the root of the penis to measure the intracavernous pressure (ICP). The CN was stimulated with a bipolar electrode (1.5 mA at 12 Hz for 60 s) by using a BL-420s Biological Functional System (Chengdu Taimeng Technology Ltd., China). The ratios of max ICP and total ICP to MAP were recorded and calculated to evaluate erectile function.

### Immunofluorescence

After functional evaluation, the penis of each eight models was collected. The penis was lifted and cut in the root. The collected tissues were washed in PBS twice. The penile mid-shaft tissues were collected for morphometry and the other portions for western blot or real-time PCR. For immunofluorescence, the collected penis and MPG fragments were cut into frozen tissue sections (5 μm), and the orientation of the slices was from dorsal to ventral. After washing with PBS three times, the sections were blocked with 3% bovine serum albumin and 0.1% Triton X-100 at room temperature for 1 h, and then, penile sections or MPG sections were incubated at 4°C against the following antibodies overnight: smooth muscle actin (SMA) (Abcam, Cambridge, United Kingdom; 1:1,000), desmin (Abcam, Cambridge, United Kingdom; 1:200), eNOS (Abcam, Cambridge, United Kingdom; 1:500), neuronal nitric oxide synthases (nNOS) (Abcam, Cambridge, United Kingdom; 1:200), nerve growth factor (NGF) (Abcam, Cambridge, United Kingdom; 1:1,000), and myelin basic protein (MBP) (Abcam, Cambridge, United Kingdom; 1:1,000). After washing thrice with PBS, the sections were incubated with daylight 488- or 556-coupled secondary antibodies (Invitrogen, San Diego, CA, United States; 1:1,000) in the dark at room temperature for 1 h, then washed three times with PBS, and the nuclei were stained with 40,6-dimidyl-2-phenylindole indole (Beyotime, Beijing, China). We evaluated the morphometry by a random and blind method. Three visual fields under a microscope were captured randomly in each specimens by a technician who was blind to the groupings. Then, a quantitative analysis of the captured photographs was made by two independent colleagues who were also blind to the grouping. The fluorescence intensities were evaluated by target IOD using ImageJ (National Institutes of Health, Bethesda, MD, United States). Finally, unblinding was made by another independent colleague who made a statistical analysis.

### Masson’s Trichrome Staining

The penile mid-shaft tissues were used for Masson’s trichrome staining. Penile tissues were washed with PBS twice, then fixed with 4% paraformaldehyde for 48 h, and embedded in paraffin before sectioning. The thickness of the section slice is 5 μm. Masson’s trichrome staining was used to distinguish the corpus cavernosum smooth muscle from collagen fibers in which smooth muscles appear red, while collagen fibers appear blue. The representative photos were captured using a digital camera, and the data analysis was carried out by Image-Pro Plus 6.0 software (Media Cybernetics, Rockville, MD).

### TUNEL Assay

The general level of apoptosis in the penile tissue was also observed by TUNEL staining using the TUNEL *in Situ* Cell Death Detection Kit (Roche) according to the manufacturer’s instruction.

### Real-Time Polymerase Chain Reaction

The other portions of penile tissue were then immediately stored in liquid nitrogen and used for western blot or real-time PCR. For real-time polymerase chain reaction, the tissues were taken out from liquid nitrogen and homogenized using a homogenizer, with a sample size of eight in each group. After extracting the total RNA from penile tissue using TRIzol reagent (Invitrogen), complementary DNA was synthesized and real-time PCR was performed using a reverse transcription kit (Roche Applied Science, Mannheim, Germany) and QuantiTect SYBR Green PCR Kit (Roche Applied Science) according to the manufacturer’s instructions, respectively. The results were analyzed using a LightCycler 480 real-time PCR system (Roche Diagnostics, Indianapolis, IN).

### Western Blot

Protein in the penile tissue was extracted by a protein extraction reagent (Beyotime, Beijing, China), which contained RIPA and phenylmethylsulfonyl fluoride. The BCA Protein Assay Kit (Beyotime, Beijing, China) was used to detect the protein concentration, and 50-μg aliquots of proteins were used for electrophoresis. After electrophoresis, the protein was transferred to the PVDF membrane and probed with the following antibodies: collagen I, collagen IV, and fibronectin (Abcam; 1: 1,000). The secondary antibody (Abcam; 1:5,000) was used in combination with the primary antibody after overnight incubation, and the bands were observed with an enhanced chemiluminescence substrate (Millipore, MA).

### Statistical Analysis

The data provided represent the mean ± standard deviation. GraphPad Prism v.7.0 (GraphPad, La Jolla, CA) was used for comparison between groups and analysis of variance. Distribution of the data was estimated by Kolmogorov–Smirnov test. The data were all normally distributed, and Newman–Keuls *post hoc* analysis was used to compare the differences between groups. The difference was statistically significant at *p* < 0.05.

## Results

### Isolation and Characterization of hGMSCs

**FIGURE 1:**
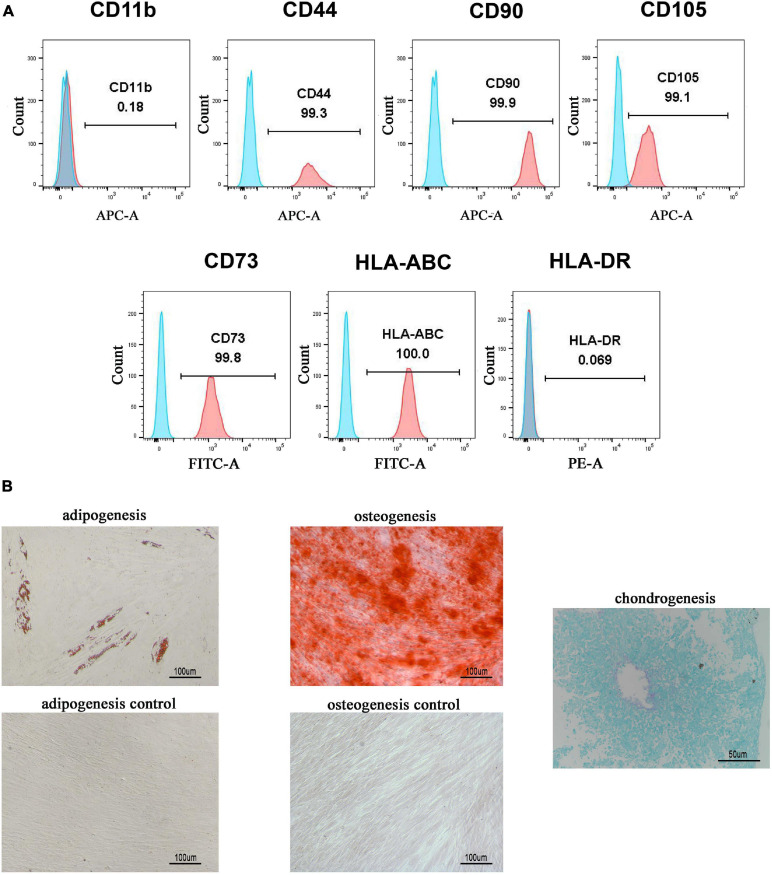
Isolation and characterization of hGMSCs. **(A)** Flow cytometry analysis of the surface marker of hGMSCs. **(B)** Adipogenic and osteogenic differentiation assays of hGMSCs. hGMSCs, human gingiva-derived mesenchymal stem cells.

hGMSCs were isolated from human gingival tissues, expanded, and passaged into third generation *in vitro* as we described previously (Chen et al., 2013; Zhang et al., 2019). A flow cytometry analysis was used to confirm that the isolated hGMSCs possessed MSC makers. The results showed that hGMSCs expressed high levels of CD44, CD90, CD105, CD73, and HLA-ABC markers and did not express CD11b or HLA-DR (Figure 1A), which confirmed its MSC characteristics. Moreover, it was also verified that hGMSCs possessed the capacity for adipogenic, osteogenic, and chondrogenic differentiation but the control cells HSFb do not (Figure 1B). For the chondrogenic differentiation assays, the HSFb could not form cartilage ball, so there was no picture presented.

### hGMSC Treatment Markedly Improves Erectile Function in CNI Rat Models

**FIGURE 2:**
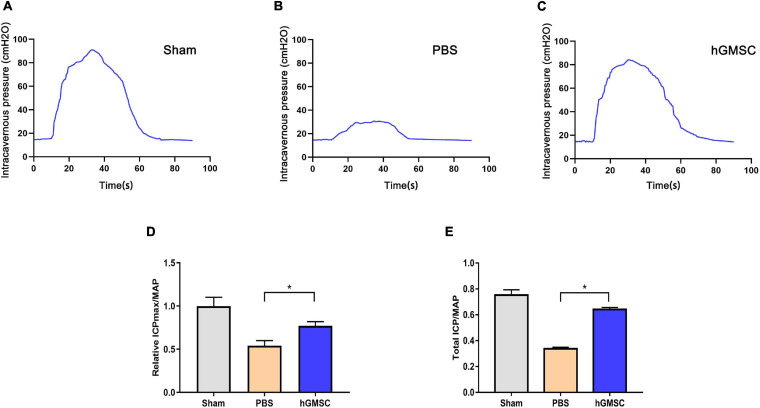
hGMSC treatment improves erectile function in CNI rat models. **(A)** Penile pressure manometry showed that hGMSC treatment significantly improves the ICP/MAP of erectile function in rats. **(B)** The quantitative results of ICPmax/MAP. **(C–E)** The quantitative results of total ICP/MAP. *p < 0.05. CNI, cavernous nerve injury; ICP, intracavernous pressure; MAP, mean arterial pressure; ICPmax, maximum ICP.

To evaluate the effects of hGMSCs on the recovery of erectile function, the rats were randomly assigned into three groups: sham operation (sham group), bilateral CNI and PBS injections (PBS group), and bilateral CNI and hGMSCs administration (hGMSC group). Two weeks after surgery and therapy, the erectile functions of CNI rat were assessed. The results showed that there were no significant differences in MAP among the three groups. However, the maximal ICP (ICPmax)/MAP and total ICP/MAP were significantly decreased in the PBS group than the sham group. Importantly, the maximal ICP (ICPmax)/MAP and total ICP/MAP were significantly improved in the hGMSC group when comparing with the PBS group, which indicated the therapeutic action of hGMSCs on CNI ED (Figures 2A–E).

### hGMSC Treatment Increases the Smooth Muscle and Endothelial Content in the Corpus Cavernosum

**FIGURE 3:**
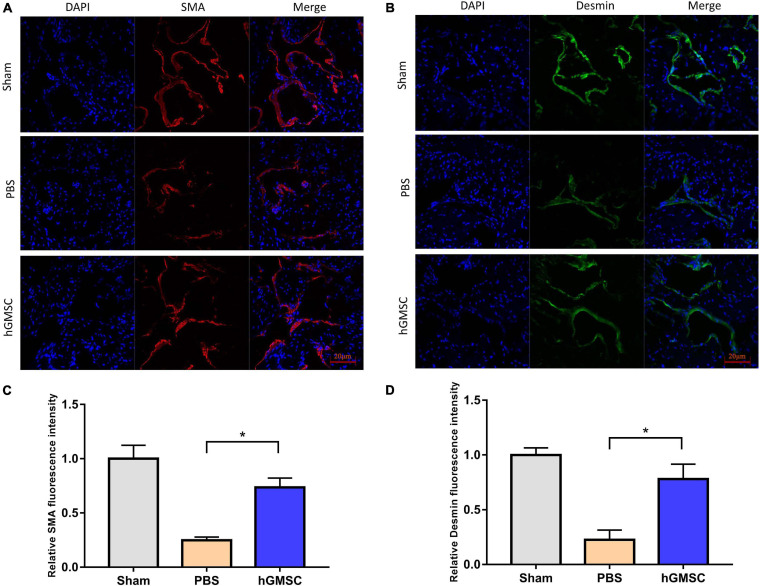
hGMSC treatment increases the smooth muscle content in the corpus cavernosum. **(A)** Immunofluorescence showed that hGMSC significantly increased the SMA content in penile tissue of rats. **(B)** The quantitative results of statistical analysis. *p < 0.05. **(C)** Immunofluorescence showed that hGMSC significantly increased the desmin content in penile tissue of rats. **(D)** The quantitative results of statistical analysis. *p < 0.05. SMA, smooth muscle actin.

**FIGURE 4:**
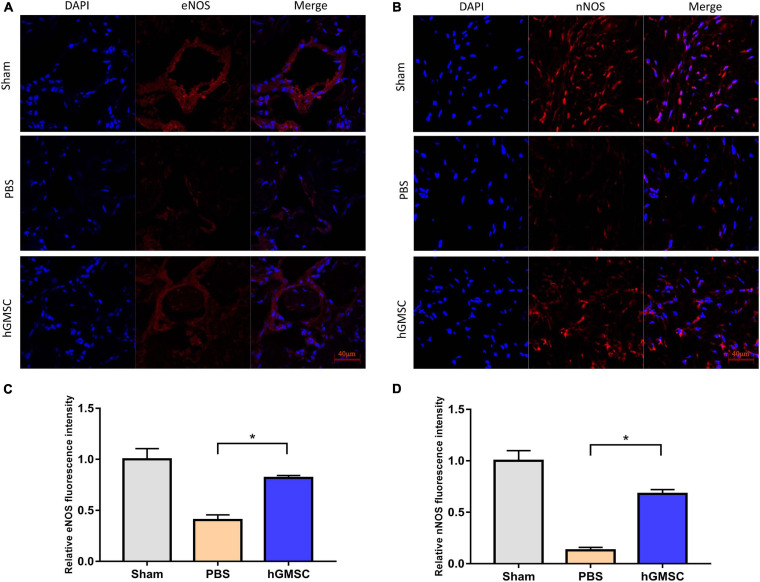
hGMSC treatment increases endothelial and neural content in the corpus cavernosum. **(A,C)** Immunofluorescence showed that hGMSC significantly increased eNOS content in penile tissues of rats. **(B,D)** Immunofluorescence showed that hGMSC significantly increased the nNOS content in penile tissues of rats. *p < 0.05. nNOS, neuronal nitric oxide synthases.

The smooth muscle and endothelial tissues are a fundamental content in penile tissue, which are vital elements for erection function and sexual performance. We then collected the penile tissues from the above rat models and assessed their expressive levels in the three groups. The immunofluorescent stainings of smooth muscle cell markers and endothelial marker were performed in the corpus cavernosums. As showed, the levels of smooth muscle cell markers, alpha-smooth muscle actin (α-SMA), and desmin were decreased in the PBS group compared with the sham group, yet rescued in the hGMSC group (Figures 3A–D). Consistently, transplantation of hGMSCs was also notably able to attenuate the decrease in eNOS expression caused by CNI (Figures 4A,C). These results indicated that an injection of hGMSCs could obviously increase the smooth muscle and endothelial content in the corpus cavernosum of CNI rats, which were all benefit for erectile function recovery.

### hGMSC Treatment Restores nNOS Expression

As known, nNOS/cGMP is a vital pathway for erectile function and nNOS is a vital molecule. Hence, the expression of nNOS in penile tissues in the three groups was further determined. Results of immunofluorescent staining showed that CNI remarkably decreased the level of nNOS in penile tissues. However, transplantation of hGMSCs significantly restored the nNOS expression when comparing with the PBS group (Figures 4B,D).

### hGMSC Treatment Attenuated Cell Apoptosis via Promoting NGF and Myelin Basic Protein Content in MPG

**FIGURE 5:**
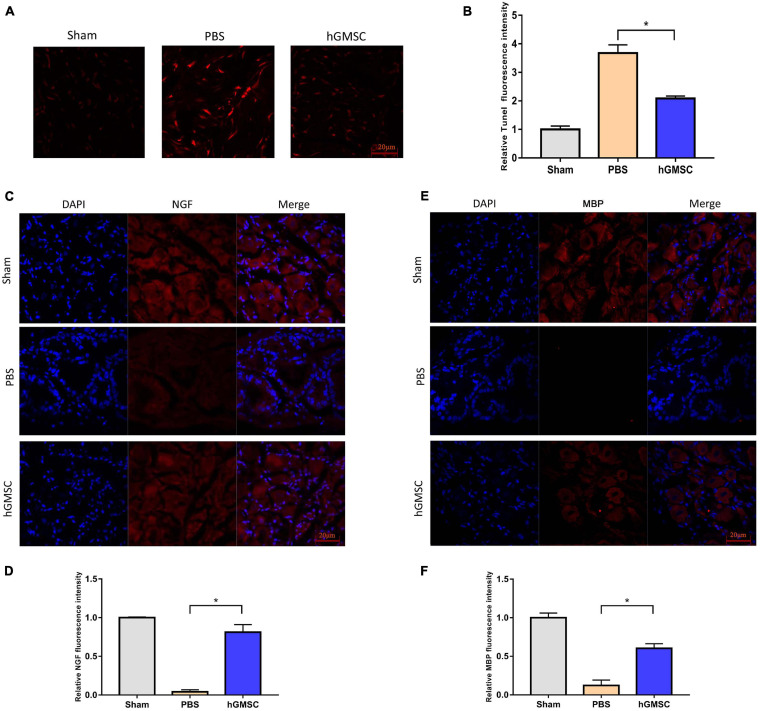
hGMSC treatment attenuates cell apoptosis via promoting NGF and MBP content in MPG. **(A)** TUNEL staining showed that hGMSC treatment significantly inhibits penile tissue apoptosis in rats. **(B)** The quantitative results of statistical analysis. *p < 0.05. **(C)** Immunofluorescence showed that hGMSC significantly increased NGF content in MPG tissues of rats. **(D)** The quantitative results of statistical analysis. *p < 0.05. **(E)** Immunofluorescence showed that hGMSC significantly increased MBP content in MPG tissues of rats. **(F)** The quantitative results of statistical analysis. *p < 0.05. NGF, nerve growth factor; MBP, myelin basic protein; MPG, major pelvic ganglia.

To further determine the cellular protective roles of hGMSCs, we performed *in vivo* TUNEL assays in penile tissues and assessed it by a laser scanning confocal microscope. The result showed that hGMSCs could effectively protect cells from apoptosis after CNI (Figures 5A,B). Furthermore, the NGF and MBP content in MPG was all increased after hGMSC treatment in CNI rat models (Figures 5C–F). Taken together, these results above demonstrated that hGMSC therapy might exert anti-apoptotic effects via promoting NGF and MBP expression.

### hGMSC Treatment Protects the Corpus Cavernosum From Fibrosis by Skewing Macrophage Polarity Toward the M2 Phenotype

**FIGURE 6:**
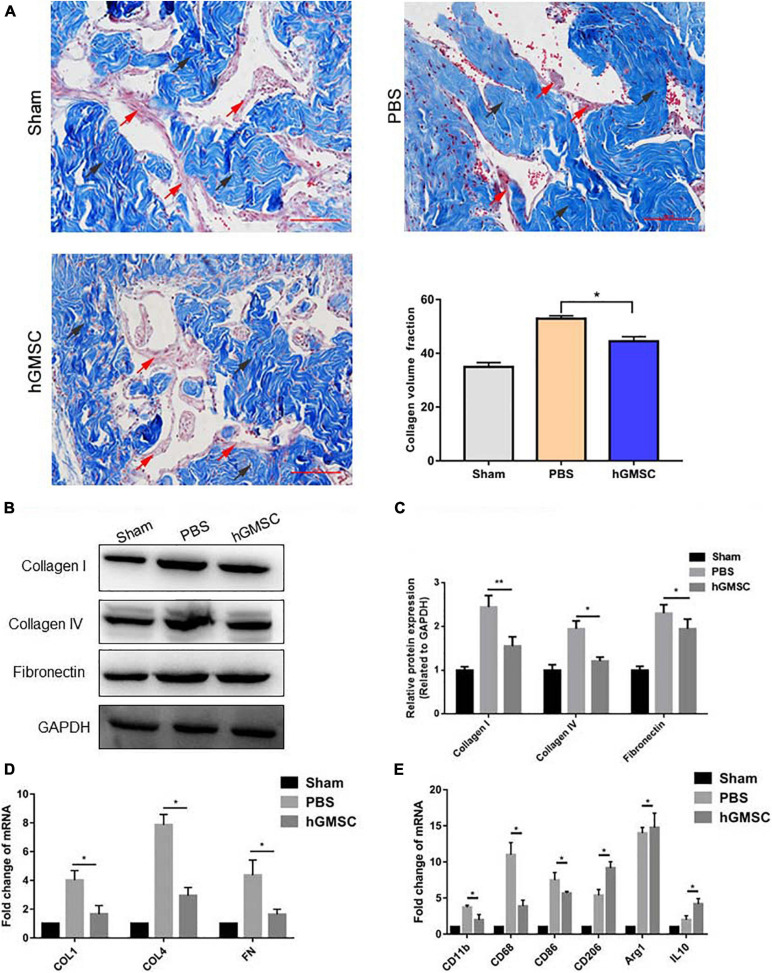
hGMSC treatment protects the corpus cavernosum from fibrosis by skewing macrophage polarity toward the M2 phenotype. **(A)** Masson’s trichrome staining indicated that hGMSC treatment significantly improves the smooth muscle/collagen ratio in penile tissues of rats. **(B,C)** Western blotting showed that hGMSC treatment significantly inhibited the expression of fibrotic markers. **(D)** qPCR indicated that hGMSC treatment significantly inhibited penile tissue fibrosis of rats. **(E)** qPCR indicated that hGMSC treatment significantly increased M1 component and decreased M2 component in penile tissues of rats. Black arrows: collagen tissues; red arrows: smooth muscle tissues. Scale bar = 100 μm, *p < 0.05, **p < 0.01.

It was well acknowledged that fibrosis of the corpus cavernosum after CNI promotes the development of ED (Urban et al., 2015). In order to evaluate the degree of fibrosis, we used Masson’s trichrome staining and found that the proportion of smooth muscle to collagen in the PBS group was significantly decreased, while hGMSC treatment showed remarkable prevention (Figure 6A). In addition, the protein and mRNA expression of fibrotic markers, such as fibronectin (FN), collagen 1 (COL 1), and collagen 4 (COL 4), which up-regulated after CNI, was decreased upon hGMSC transplantation (Figures 6B–E).

We then detected M1 macrophage markers, M2 macrophage markers, and chemokines in the penile tissues by qPCR. The results showed that hGMSC treatment significantly reduces the expression of M1 macrophage markers (CD11b, CD68, and CD86) but enhances the expression of M2 macrophage markers (CD208, Arg1, and IL10), which demonstrates that hGMSC treatment might skew macrophage polarity from M1 toward M2 phenotype, subsequently protecting the corpus cavernosum from fibrosis (Figure 6E).

## Discussion

ED occurs after pelvic surgery frequently, which is due to intraoperative damage of the pelvic cavernous nerve. Unfortunately, traditional treatments, such as PDE5 inhibitors, is not well responsive for neuropathic ED. Many studies have been focusing on the development of strategies for controlling and improving this specific kind of ED, but none is successful so far. Herein, we demonstrated that hGMSCs, an optimized substitution for MSCs derived from other sources, could protect against CNI-induced penile atrophy, apoptosis, and fibrosis by skewing macrophage polarity toward the M2 phenotype, which may provide an innovative strategy to treat CNI ED.

Given their ability to modulate inflammation, paracrine function, and low immunogenicity, a cell therapy based on MSCs has been considered as the most hopeful approach for tissue injury, repair, and regeneration (Trounson and McDonald, 2015). Previously, we focused on exploring the more promising approaches to treat CNI ED. Inspiringly, we found that not only a modification of adipose-derived MSCs (adMSCs) by co-overexpression of VEGF and GDNF but also a combined transplantation of MSCs and endothelial progenitor cells could significantly restore CNI ED partially via exosomes (Fang et al., 2018; Ouyang et al., 2018; Fang S. B. et al., 2020; Yang et al., 2020). Indeed, exosomes mainly contribute to functional activities of MSC (Fang J. et al., 2020). Moreover, transplantation of induced pluripotent stem cell-derived mesenchymal stem cells (iMSCs) also significantly improved ED via paracrine factors (Chen et al., 2019). Compared with the more invasive procedure to obtain adMSCs and higher potential tumorigenic risk of iMSCs (Zhang et al., 2016; Huang et al., 2018a,b), we used hGMSCs and explored their therapeutic effects in CNI ED in this study. As we know, hGMSCs exhibited higher viability and stability and thus might be an optimized substitution for MSCs derived from other sources in therapy. More recently, we have reported these cells are highly safe in various animal safety experiments (Luo X. Y. et al., 2019). Similarly, transplantation of hGMSCs in a rat model of CNI exhibited favorable therapeutic effects by improving erectile function measured by ICPmax/MAP and total ICP/MAP, increasing the smooth muscle and endothelial integrity in the corpus cavernosum, as well as attenuating apoptosis and fibrosis in penile tissues. Mechanically, hGMSC treatment increased paracrine factors like NGF and promoted macrophage polarization toward an anti-inflammatory M2 phenotype. Thus, these studies suggested that hGMSC might be a promising approach for CNI ED therapy.

A previous study has revealed that CNI could lead to progressive fibrosis of the corpus cavernosum, subsequently promoting the progression of ED (Ferrini et al., 2009). In fact, the development of pathological fibrosis was caused by chronic or dysregulated wound healing response, impairing normal tissue function and ultimately leading to organ failure (Wynn and Ramalingam, 2012). After CNI, the fibrosis response of the penile cavernous cavernosum was acute at 1 week, then turned to chronic gradually after 12 weeks (Song et al., 2015). Moreover, penile cavernous fibrosis might still continue to progress, even CN was repaired successfully. Large amounts of studies have previously proven that resident tissue macrophages are key regulators of tissue repair and fibrosis (Wynn and Vannella, 2016). Matsui et al. (2017) previously reported that M1 macrophages were predominantly recruited to the MPG after CNI. Numerous researches had revealed that inflammatory resident tissue macrophages are key regulators of tissue fibrosis (Wynn and Barron, 2010). Therein, two types of macrophages according to distinct surface markers, M1 (classically activated macrophages) and M2 (alternatively activated macrophages), were involved in organ fibrosis. M1 macrophages, characterized by the expression of CD68^+^CD86^+^ and secretion of pro-inflammatory cytokines (Luo and Zheng, 2016), were described as the pro-inflammatory type. M1 macrophages participate in the starting point of pro-fibrotic process, via promoting the recruitment and proliferation of fibrocytes, as well as EMT/EndoMT into myofibroblasts (Liu et al., 2008; Braga et al., 2015), while M2 (CD206^+^Arg1^+^) macrophages were recognized to exert quite the opposite function: secretion of anti-inflammatory cytokines like IL-10 and participation in the repair of damaged and fibrotic tissues (Ley, 2017).

It was previously reported that M1 macrophage infiltration was increased in MPG after CNI, which was associated with progression of ED (Matsui et al., 2017). To our knowledge, our study was the first to explore the effect of hGMSCs on macrophage polarity in penile tissue after CNI. We found that hGMSC treatment could skew macrophage polarity from the M1 toward M2 anti-inflammatory phenotype and release IL-10, which could be considered as one potential mechanism alleviating penile fibrosis. Consistently, our previous study had revealed that hGMSC treatment markedly reduced inflammatory macrophage activation, converting inflammatory to anti-inflammatory phenotype *in vitro*, which further indicated that hGMSCs could affect the polarization and activation of macrophages (Zhang et al., 2018). In addition, Luo X. Y. et al. (2019) also found that M2 macrophages could be activated and elevate the expression of IL-10 and MMP13 after transplantation of BM-MSCs, which played synergistic roles in attenuating liver fibrosis. However, the recruited macrophages in bleomycin-induced fibrotic lungs were mainly M2 macrophages, which promoted myofibroblast differentiation (Hou et al., 2018). Taken together, given the somewhat contradictory functional roles of M2 macrophage during fibrosis, tissue macrophages were considered to play distinct roles in different pathologic environments and specific stages of injury and repair (Byrne et al., 2016).

There are also some limitations to our study. Firstly, we used rats as CNI ED models to explore the therapeutic effects of hGMSCs, which may not be a good reflection of humans. Further studies using hGMSCs on mammal ED models would be helpful for a better understanding of the therapeutic actions of hGMSCs. Secondly, the functions of hGMSCs were only evaluated in 2 weeks after transplantation, which could better reflect the early treatment effect of hGMSCs on CNI ED, but the long-term therapeutic effects, especially the anti-fibrotic roles, should be determined in the future. Thirdly, the underlying mechanisms of how hGMSCs influenced macrophage polarization were not fully explored in our study, which may impede the formation of an optimal therapeutic method for CNI ED. Last but not the least, we did not label and trace the hGMSCs *in vivo*. Our future study will further determine whether hGMSCs have a regeneration ability to directly repair the injured tissues to restore the function.

When assessing the clinical perspective of regenerative therapy methods in patients with oncology, we hold the opinion that the present exploration is a fundamental research and might possess a promising clinical application prospect. On the one hand, it has been proved that MSCs hold extremely limited possible tumorigenicity and there is no tumorigenesis report in its clinical applications. On the other hand, there are some preliminary clinical trials that applied MSCs in neurological ED patients and showed positive results. Hence, we hold the opinion that human gingiva-derived MSCs would be a promising therapeutic cell for ED patients who received radical prostatectomy and rectectomy due to pelvic oncology and need further clinical trials in the future.

## Conclusion

In conclusion, our study first revealed the protective functions of hGMSCs against CNI-induced penile atrophy, apoptosis, as well as fibrosis, owing to the function of skewing macrophage polarity toward the M2 anti-inflammatory phenotype. Therefore, transplantation of hGMSCs may be a promising strategy to treat the CNI ED in future clinical applications.

## Data Availability Statement

The original contributions presented in the study are included in the article/supplementary material, further inquiries can be directed to the corresponding author/s.

## Ethics Statement

The animal study was reviewed and approved by the Animal Care and Use Subcommittee of The Third Affiliated Hospital of Sun Yat-sen University.

## Author Contributions

HW and SZ contributed to the conception of the study. JW performed the experiments and wrote the manuscript. ZC and FZ contributed to analysis and manuscript preparation. WY and XO performed the data analyses. XM helped to constructive discussions. All authors contributed to manuscript revision as well as read and approved the submitted version.

## Conflict of Interest

The authors declare that the research was conducted in the absence of any commercial or financial relationships that could be construed as a potential conflict of interest.
